# A Novel Green In Situ Amine-Functionalized Aerogel UiO-66-NH_2_/TOCNF for the Removal of Azo Anionic Dyes

**DOI:** 10.3390/gels11050365

**Published:** 2025-05-15

**Authors:** Rabia Amen, Islam Elsayed, Yunsang Kim, Gregory T. Schueneman, Emad M. El-Giar, El Barbary Hassan

**Affiliations:** 1 Department of Sustainable Bioproducts, Mississippi State University, P.O. Box 9820, Mississippi State, MS 39762, USA; ra1070@msstate.edu (R.A.); iae10@msstate.edu (I.E.); ysk13@msstate.edu (Y.K.); 2 Department of Chemistry, Faculty of Science, Damietta University, New Damietta 34517, Egypt; 3 USDA Forest Service, Forest Products Laboratory, Madison, WI 53726, USA; gregory.t.schueneman@usda.gov; 4 School of Sciences, University of Louisiana at Monroe, Monroe, LA 71209, USA; elgiar@ulm.edu

**Keywords:** Orange II, Congo red, zirconium, MOF, nanocellulose, wastewater

## Abstract

UiO-66-NH_2_ is a metal–organic framework (MOF) with open metal sites, making it a promising candidate for adsorption and catalysis. However, the powdery texture of MOFs and the use of toxic solvents during synthesis limit their application. A novel solution to this issue is to create a layered porous composite by encasing the MOF within a flexible and structurally robust aerogel substrate using safe, eco-friendly, and green solvents such as ethanol. The fibrous MOF aerogels, characterized by a desirable macroscopic shape of cylindrical block and hierarchical porosity, were synthesized by two approaches: in situ growth of amine-functionalized UiO-66-NH_2_ crystals on a TEMPO-oxidized cellulose nanofiber (TOCNF) and ex situ crosslinking of UiO-66-NH_2_ crystals onto a TOCNF network to form UiO-66-NH_2_/TOCNF. The incorporation of MOF into the cellulose nanofibrils via the in situ method reduces their aggregation potential, alters the nucleation/growth balance to produce smaller MOF crystals, and enhances mechanical flexibility, as evidenced by SEM images. The three adsorbents, including UiO-66-NH_2_, ex situ UiO-66-NH_2_/TOCNF, and in situ UiO-66-NH_2_/TOCNF, were synthesized and used in this study. The effects of pH, time, temperature, and initial concentration were studied. A maximum adsorption capacity (Qmax) of 549.45 mg/g for Congo Red (CR) and 171.23 mg/g for Orange II (ORII) was observed at pH 6, using 10 mg of in situ UiO-66-NH_2_/TOCNF at 40 °C with a contact time of 75 min for CR and 2 h for ORII. The adsorption of both dyes primarily occurs through monolayer chemisorption on the in situ UiO-66-NH_2_/TOCNF. The main removal mechanisms were hydrogen bonding and surface complexation. The noteworthy adsorption capacity of in situ UiO-66-NH_2_/TOCNF coupled with environment-friendly fabrication techniques indicates its potential applications on a large scale in real wastewater systems.

## 1. Introduction

The development during the industrial era has accelerated water contamination with pharmaceuticals, heavy metals, and synthetic dyes [1]. Among them, azo anionic dyes, characterized by one or more azo (-N=N-) bonds, cause significant environmental and health hazards due to their high water solubility, chemical stability, and potential carcinogenicity [2]. Approximately 17% to 20% of global water contamination originates from colored wastewater [3]. Congo Red is an azo dye that is classified as cytotoxic and banned in most countries but still used by histologists to identify amyloid. It has toxic, carcinogenic, and mutagenic effects on living organisms. Orange II is an azo dye used in the printing and clothing industry that can harm the brain, kidneys, and liver. It cannot be removed from water by UV, acid–base, or biological treatments.

Photocatalytic degradation, chemical precipitation, ion exchange, adsorption, oxidation, membrane separation, and various other methods have been widely employed to remove dyes from wastewater [4]. These techniques are costly, have high waste byproduct generation, and are ineffective for industrial wastewater due to its complex chemistry. The adsorption is a cost-effective, simple, and safer technique for industrial wastewater treatment [5]. Various adsorbent materials are used for water treatment, including carbon-based adsorbents, biosorbents, and minerals. However, they have poor regeneration potential and efficiency, which makes their use ineffective and expensive. The biosorbents are mechanically unstable and have fouling characteristics, which make them ineffective for water treatment. The highly effective adsorption of dyes relies on the availability of large specific surface areas and higher porosity of the material [6]. Metal–organic frameworks (MOFs) have the potential to revolutionize various fields, such as gas adsorption, capacitors, and sensors, due to their excellent specific surface area and pore volume compared to other porous materials like metal oxides, silica, and mesoporous materials [7,8].

The MOF UiO-66-NH_2_, a zirconium-based porous crystalline material, is less expensive than other MOFs due to its simple fabrication process and the availability of precursors such as zirconium and ammonia [9]. Compared to the solvo-thermal method, aqueous solution-based MOFs are also environmentally friendly, reducing the overall carbon footprint by 91% and the fabrication cost of UiO-66-NH_2_ by 84% [10]. Although MOFs are widely used in wastewater treatment, they have limitations due to their nano-powder nature and complex structure, which can lead to fouling and loss during reuse and recycling, ultimately reducing recyclability [4]. Supporting MOFs on a suitable substrate appears to be an effective way to enhance their performance and recyclability [11]. Biodegradable materials such as aerogels, cellulose, hydrogels, and membranes are now regarded as outstanding support materials for MOFs [12,13]. MOFs such as UiO-66 have received attention for their large surface area, variable pore size, and exceptional chemical stability [14]. Cellulose-based nanomaterials, particularly 2,2,6,6-tetramethylpiperidine-1-oxyl radical (TEMPO)-oxidized cellulose nanofibers (TOCNF), have emerged as environmentally friendly, biodegradable, and structurally robust platforms for aerogel fabrication [15]. These CNFs exhibit excellent mechanical properties, a high aspect ratio (200–500), and a narrow width (3–5 nm) [16,17]. Due to the abundance of carboxylate groups (-COO-) on TOCNF surfaces, they can readily cross-link with functional groups on MOFs [18]. Thus, there is potential to create highly effective adsorption materials via the combination of UiO-66 MOF and TOCNF.

The concept of organometallic catalysts supported on organic materials has been explored previously. Zhu et al. incorporated ZIF-8 crystals into TEMPO-oxidized cellulose nanofibrils to produce shapeable and flexible aerogels, which exhibited a high adsorption capacity for various dyes, including rhodamine B (Q_max_ = 81 mg/g), methyl blue (q_e_ = 13 mg/g), methyl orange (q_e_ = 4 mg/g), methyl violet (q_e_ = 27 mg/g), and methylene blue (q_e_ = 24 mg/g) [19]. A well-dispersed mixed matrix membrane was developed by incorporating wet UiO-66-NH_2_ into a poly(vinylidene fluoride) (PVDF) matrix for the removal of Rhodamine B (RhB) and Congo Red (CR) from aqueous solutions, achieving maximum adsorption capacities of 25.29 mg/g and 16.50 mg/g, respectively [20]. Wang et al. prepared composite UiO-66/nanocellulose aerogels with hierarchical porosity and low density using a self-crosslinking method, successfully achieving adsorption of both anionic methyl orange (MO, q_max_ = 71.7 mg/g) and cationic methylene blue (MB, q_max_ = 51.8 mg/g) [21]. UiO-66 and UiO-66-NH_2_ MOFs were prepared for methylene blue (MB) adsorption and methyl orange (MO). The highest adsorption capacities were 90.88 mg/g and 96.45 mg/g for MB and 39.42 mg/g and 28.97 mg/g for MO, respectively [22]. UiO-66-NH_2_@HTC composites were synthesized by incorporating hydrothermal carbon (HTC) into the UiO-66-NH_2_ framework for the adsorption of methylene blue (MB) and Congo red (CR), achieving maximum adsorption capacities of 263.1 mg/g and 277.77 mg/g, respectively [23].

The synthetic materials and solvents typically used in MOF synthesis pose various environmental issues. The toxic solvents specifically used in solvothermal MOF synthesis techniques include N,N-dimethylformamide (DMF), dimethylacetamide (DMA), tetrahydrofuran (THF), and dichloromethane (DCM), among others [24,25,26]. The world seeks environmentally friendly systems; therefore, selecting a green synthesis method to fabricate MOF is crucial [27]. Ethanol is an environmentally friendly, renewable, and relatively nontoxic alternative to toxic solvents, and it readily dissolves metal salts and organic linkers [28,29,30]. It is produced through the fermentation of plant materials and can be metabolized by the human body [31,32]. Additionally, the 5-aminoisophthalic acid is a safer alternative compared to other ligands [33,34]. From a commercial perspective, the processes and materials involved in MOF synthesis should be inexpensive, environmentally friendly, recyclable, consume less energy, and produce minimal waste.

Herein, we investigate a novel in situ and ex situ UiO-66-NH_2_/TOCNF fabricated in ethanol as a green solvent and tested for the removal of two anionic azo dyes, Orange II (ORII) and Congo Red (CR), from synthetic wastewater. The effects of pH, time, and initial concentration were also examined. Thermodynamic and regeneration studies were conducted to evaluate the stability and adsorption potential of the adsorbate under different conditions.

## 2. Results and Discussion

### 2.1. Characterization of UiO-66-NH_2_/TOCNF Adsorbents

**Figure 1:**
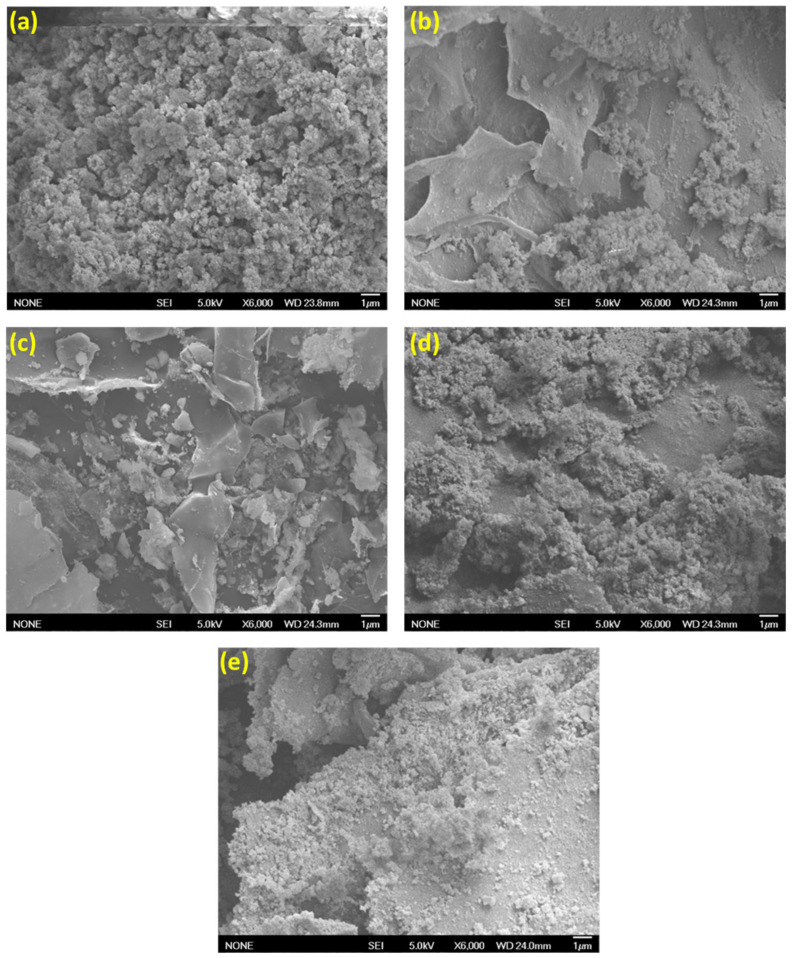
Fe-SEM micrographs of (**a**) UiO-66-NH_2_, (**b**) in situ UiO-66-NH_2_/TOCNF, (**c**) ex situ UiO-66-NH_2_/TOCNF, (**d**) in situ UiO-66-NH_2_/TOCNF after CR adsorption, and (**e**) in situ UiO-66-NH_2_/TOCNF after ORII adsorption.

**Figure 2:**
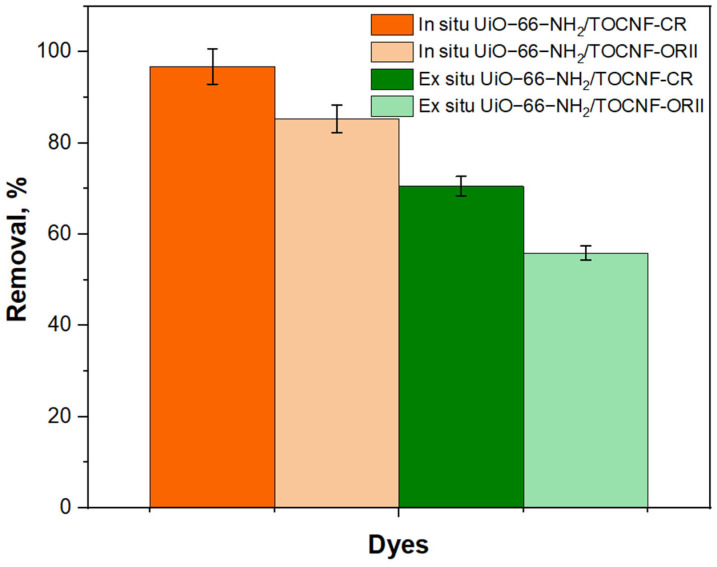
Removal % of CR and ORII using in situ UiO-66-NH_2_/TOCNF and ex situ UiO-66-NH_2_/TOCNF.

Adsorbents were fabricated from UiO-66-NH_2_ and in situ UiO-66-NH_2_/TOCNF, where the MOF was formed on aqueously dispersed TOCNF; then, an aerogel was produced. Additionally, ex situ UiO-66-NH_2_/TOCNF involved adding preformed UiO-66-NH_2_ to aqueously dispersed TOCNF, which was then formed into an aerogel. The morphologies of UiO-66-NH_2_, ex situ UiO-66-NH_2_/TOCNF, and in situ UiO-66-NH_2_/TOCNF were imaged with SEM, Figure 1. The UiO-66-NH_2_ (Figure 1a) resembles a cluster of amorphous MOFs due to the absence of a general long-range order. The micrograph of in situ UiO-66-NH_2_/TOCNF adsorbent (Figure 1b) reveals that the UiO-66-NH_2_ particles are uniformly distributed throughout the aerogel, leading to possible structural durability and strong interfacial associations. The distribution and connectivity of particles indicate rapid nucleation and generation of UiO-66-NH_2_ on TOCNF. The ex situ aerogel (Figure 1c) exhibited MOF agglomeration and weak interfacial connectivity as evidenced by delocalization of MOF clusters from the TOCNF. The in situ growth of MOF is more suitable due to enhanced dispersal and connection throughout the structure of the aerogel [35,36]. The growth of MOFs can be observed both on and within the in situ UiO-66-NH_2_/TOCNF surface (Figure 1b). The MOFs can only be observed on the exterior surface of ex situ UiO-66-NH_2_/TOCNF, which exhibited lower adsorption in initial trials (42.9% for CR and 29.8% for ORII) compared to in situ UiO-66-NH_2_/TOCNF (96.8% for CR and 85.3% for ORII) as shown in Figure 2. The low adsorption capacity of the ex situ UiO-66-NH_2_/TOCNF may be attributed to the presence of MOF just on the surface (Figure 1c). The SEM images of in situ UiO-66-NH_2_/TOCNF after CR and ORII adsorption (Figure 1d and e, respectively) clearly show a rougher surface. The surface of the in situ UiO-66-NH_2_/TOCNF remains stable after adsorption, and the dye particles are successfully captured by the in situ UiO-66-NH_2_/TOCNF.

**Figure 3:**
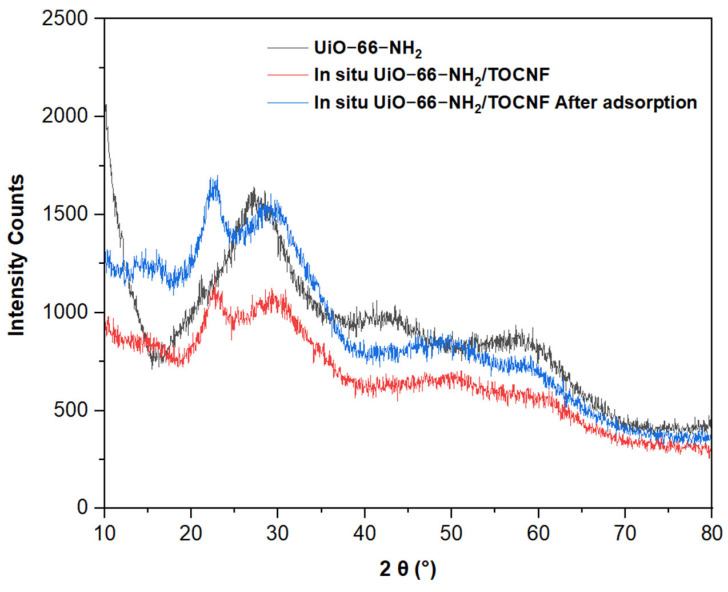
XRD of UiO-66-NH_2_ and in situ UiO-66-NH_2_/TOCNF before and after adsorption.

The XRD of in situ UiO-66-NH_2_/TOCNF exhibits prominent peaks, indicating a crystalline structure with high intensity, which suggests a high degree of crystallinity (Figure 3). The reduced peak intensities and broadened peaks of in situ UiO-66-NH_2_/TOCNF compared to UiO-66-NH_2_ may be attributed to its linkage with a TEMPO-CNF [37]. The two broad diffraction peaks at 2θ of 15.7 and 22.3°, which are attributed to the cellulose I crystalline structure; TEMPO oxidation treatment does not influence the crystalline structure of cellulose [16,38]. The peak structure of ORII-adsorbed in situ UiO-66-NH_2_/TOCNF resembles that of the original in situ UiO-66-NH_2_/TOCNF, indicating minimal structural alteration and suggesting robust reusability potential [39].

**Figure 4:**
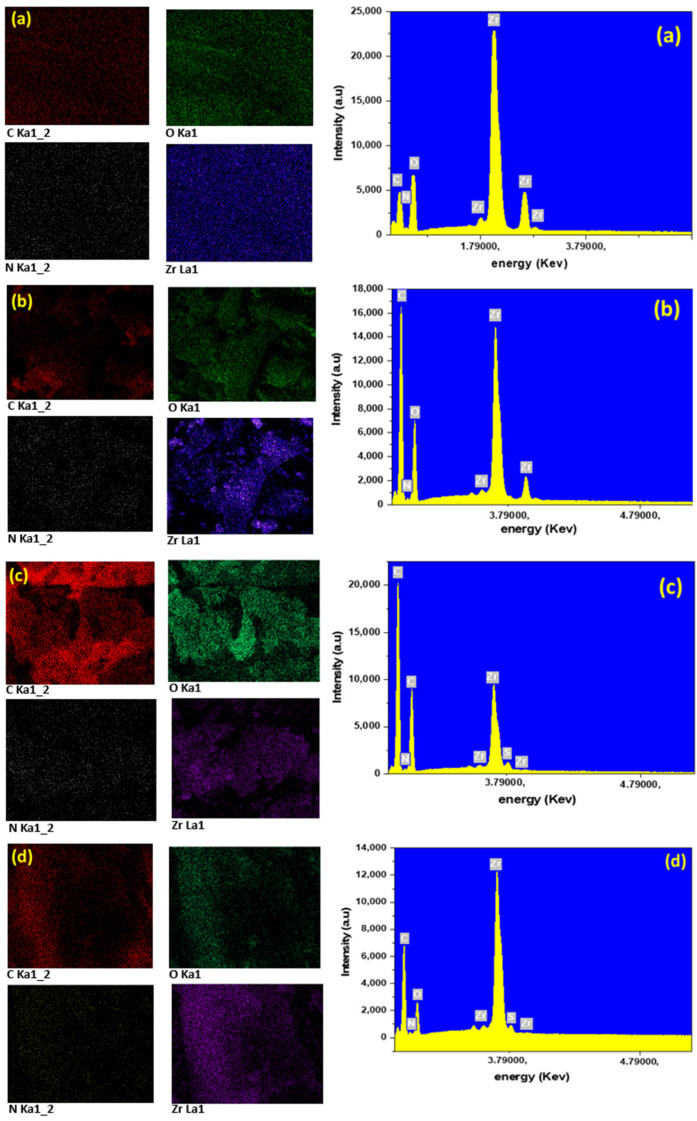
EDS atomic composition maps (**left**) and x-ray emission spectra (**right**) of (**a**) UiO-66-NH_2_, (**b**) in situ UiO-66-NH_2_/TOCNF, (**c**) ex situ UiO-66-NH_2_/TOCNF after CR adsorption, and (**d**) in situ UiO-66-NH_2_/TOCNF after ORII adsorption.

We have employed SEM to examine the morphology of UiO-66-NH_2_, in situ UiO-66-NH_2_/TOCNF, ex situ UiO-66-NH_2_/TOCNF, as well as in situ UiO-66-NH_2_/TOCNF after dye adsorption, Figure 1. Conducting scanning energy dispersive spectroscopy (EDS) within the SEM allows the collection of X-ray emission spectra and atomic composition maps. X-ray emission spectra on the right side of Figure 4a–d show the presence of Zr, C, O, and N in all adsorbents. The presence of nitrogen confirms the presence of amine groups of the organic linker inside the MOF (Figure 4a,b). The presence of sulfur in Figure 4c,d indicates the presence of the sulfonic group of both dyes after adsorption. The peak of Zr after adsorption is slightly reduced (Figure 4c,d) but not eliminated, demonstrating the stability of MOFs after adsorption.

The specific surface areas of UiO-66-NH_2_, in situ UiO-66-NH_2_/TOCNF, and ex situ UiO-66-NH_2_/TOCNF are 60.417, 145.670, and 26.701 m^2^/g, respectively (Table 1). The high surface area of in situ UiO-66-NH_2_/TOCNF, compared to UiO-66-NH_2_ and ex situ UiO-66-NH_2_/TOCNF, may be attributed to the growth of MOFs within the aerogel during the in situ synthesis, which prevents agglomeration. The low surface area and adsorption capacity of ex situ UiO-66-NH_2_/TOCNF appear to be correlated for both dyes. The low surface area may result from the lack of impregnation into and agglomeration on the already prepared MOFs on the surface of the nanocellulose aerogel during the ex situ fabrication [19].

**Figure 5:**
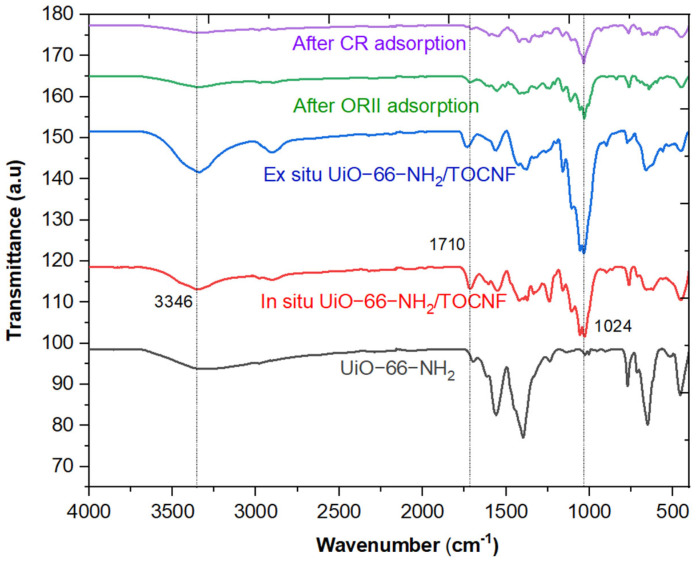
FTIR Spectra of UiO-66-NH_2_, in situ UiO-66-NH_2_/TOCNF, ex situ UiO-66-NH_2_/TOCNF, in situ UiO-66-NH_2_/TOCNF after CR adsorption, and in situ UiO-66-NH_2_/TOCNF after ORII adsorption.

FTIR analysis has been conducted to investigate the successful modification and adsorption of dyes (Figure 5). The peak observed at 1710 cm^−1^ indicates the stretching vibrations of the carbonyl group in the carboxylate (–COOH) moieties present in UiO-66-NH_2_ and in situ UiO-66-NH_2_/TOCNF [22,40]. The asymmetric stretching vibration detected at 1445 cm^−1^ is attributed to the O=C=O group, while the peak at 1160 cm^−1^ corresponds to the symmetric bending vibration of the O–C–O group [22,41]. The vibration peak at 1000 cm^−1^ that is present in in-situ UiO-66-NH_2_/TOCNF suggests hydrogen bond association between the -NH_2_ group of UiO-66-NH_2_ and the -OH or -COOH groups of CNF-COOH [42]. The vibration peaks observed at 1558, 1445, and 1237 cm^−1^ are attributed to C=O, C–N, and C–O functional groups, respectively [22]. The broad vibrational peak observed at 3346 cm^−1^ in UiO-66-NH_2_, in situ UiO-66-NH_2_/TOCNF before and after dye removal, and ex situ UiO-66-NH_2_/TOCNF MOFs indicates the stretching vibrations of the amino group and hydroxyl groups [43]. The stretching vibration peaks located at 650 and 456 cm^−1^ correspond to Zr–O and Zr_6_O_4_ clusters, respectively, as observed in all spectra [44]. The S=O is responsible for vibration peaks between 1059 and 1172 cm^−1^ in in situ UiO-66-NH_2_/TOCNF after the removal of ORII and CR [45].

**Figure 6:**
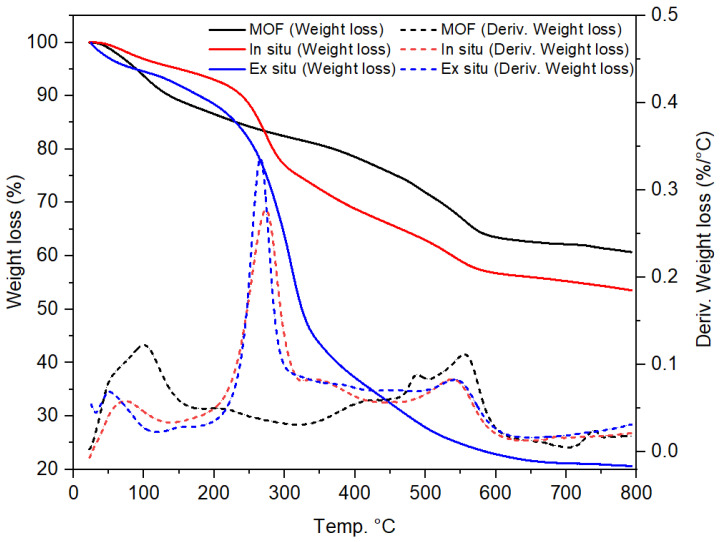
TGA-DTG thermograph of UiO-66-NH_2_, in situ UiO-66-NH_2_/TOCNF, and ex situ UiO-66-NH_2_/TOCNF.

TGA analysis was conducted to assess the decomposition behavior and stability of adsorbents (Figure 6). Three significant weight losses are observed in all samples. The first significant weight loss occurs around 100 °C, which can be attributed to the loss of water molecules [46]. Significant thermal degradation occurred between 200 and 350 °C in the second stage, resulting in the breaking of glycosidic linkages in the nano-cellulose via deamination, dehydration, and rearrangement, and a breakdown of UiO-66-NH_2_ crystalline framework [47,48]. The ex situ UiO-66-NH_2_/TOCNF exhibited distinct weight loss from 250 to 600 °C in a similar fashion to TOCNF alone. This significant weight loss suggests a weaker interaction between UiO-66-NH_2_ and TOCNF at the molecular level. The third weight loss in MOF, occurring around 203 °C, can be attributed to the decomposition of the material. The marked difference in thermal degradation behavior between the in situ and ex situ fabrication methods may indicate that an intimately mixed MOF–TOCNF composite structure was formed by the in situ process.

### 2.2. Adsorption Studies

#### 2.2.1. Effect of pH

**Figure 7:**
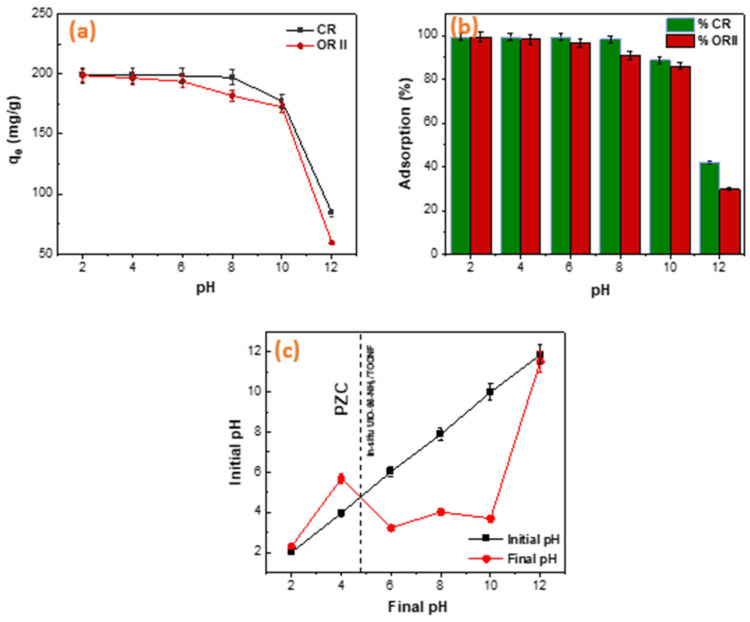
(**a**,**b**) Influence of pH on the removal performance of CR and ORII and (**c**) PZC of in situ UiO-66-NH_2_/TOCNF.

A crucial aspect of the adsorption process is the solution’s pH. The pH significantly influences chemisorption, which in turn affects the surface charge of both the adsorbent and the adsorbate. Looking at the adsorption capacity as a function of pH, the maximum adsorption of 198.87 mg/g and 198.77 mg/g is observed for the in situ adsorbent at pH 2 for CR and ORII dyes, respectively (Figure 7a). Here, we focus our discussion on the in situ adsorbent due to its significantly higher capacity. The adsorption capacity for both dyes begins to decrease at a pH of 5, which is consistent with the PZC of in situ UiO-66-NH_2_/TOCNF detected at pH = 4.8 (Figure 7c). Therefore, at pH values above 4.8, the surface of in situ UiO-66-NH_2_/TOCNF becomes negatively charged, which leads to a decrease in the adsorption of both anionic dyes due to electrostatic repulsion. From pH 6 to pH 12, the adsorption capacity of CR and ORII fell from 198.554 to 84.247 mg/g and from 193.6 to 59.68 mg/g, respectively (Figure 7a). The reduction in dye adsorption from 98% to 39.0% and 34.0% for CR and ORII, respectively, may be due to an increase in the concentration of OH species, which compete with anionic dyes for adsorption sites [49,50]. The sulfonated groups of the dye molecules in solution dissociate when the pH increases, generating anions in the solution. This aqueous solution exhibits a strong affinity for the protonated adsorbent, thereby enhancing the adsorption of both dyes.

#### 2.2.2. Adsorption Kinetics

**Figure 8:**
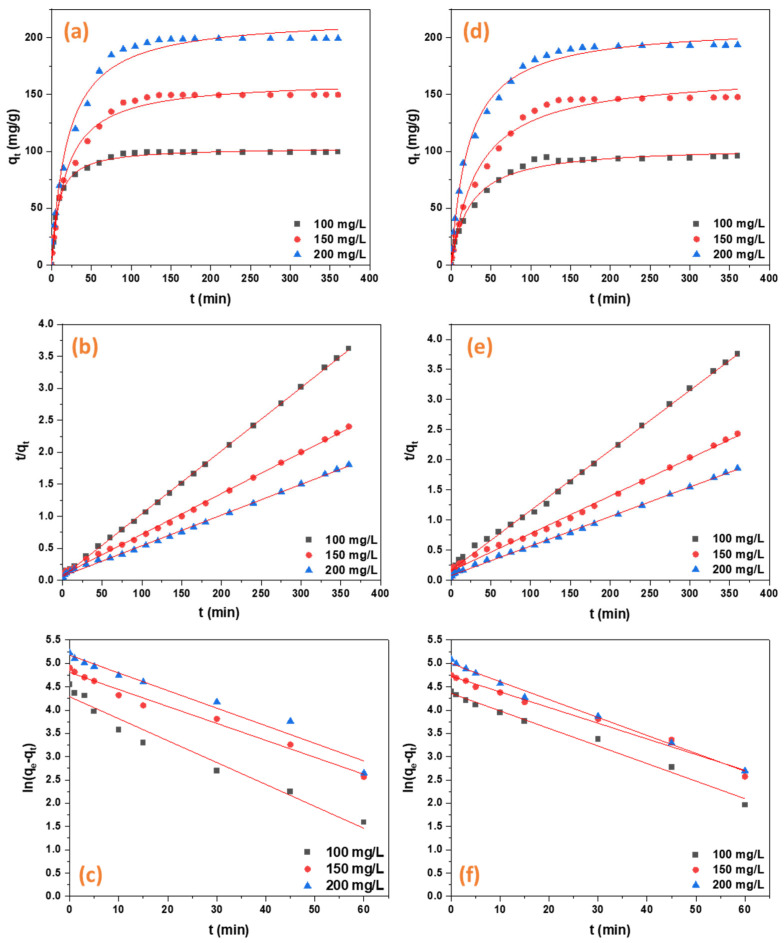
The adsorption kinetics of CR and ORII. (**a**) CR Pseudo−second order non−linear fit; (**b**) CR Pseudo−second order linear fit; (**c**) CR Pseudo−first order linear fit; (**d**) ORII Pseudo−second order non−linear; (**e**) ORII Pseudo−second order linear fit; (**f**) ORII Pseudo−first order linear fit.

The maximum adsorption percentages of 98.7% and 97.7% are exhibited by in situ UiO-66-NH_2_/TOCNF for CR and ORII, respectively. Besides providing mechanical versatility and additional porosity for the resulting MOF aerogels, the nanofibrous substrate also altered the relative nucleation and growth, producing smaller MOF crystals, thereby further reducing the likelihood of their aggregation [19]. Adsorption kinetics behavior offers valuable insights into the adsorption mechanisms and rate-limiting steps, including mass transport processes, chemical reactions, and diffusion [51]. The R^2^ value of the pseudo-second order model for both ORII and CR is greater than that of the pseudo-first order model, indicating that the data for both dyes fit the pseudo-second order model well, Table 2. The pseudo-second-order linear model is the best fit for the ORII and CR data. The functional groups of the adsorbate and adsorbents played a role in the rate-determining step of chemisorption [52]. The Qmax values for ORII and CR are 171.23 mg/g and 549.45 mg/g, respectively (Figure 8a–f, Table 2). A more precise description of the cellulose aerogel’s adsorption behavior is assumed to be achievable using the Pseudo-second order kinetic model, which suggests that chemisorption is likely the primary mechanism for both dyes, involving initial rapid diffusion into pores followed by the occupation of adsorption sites in a step-by-step fashion [53].

#### 2.2.3. Adsorption Isotherms

**Figure 9:**
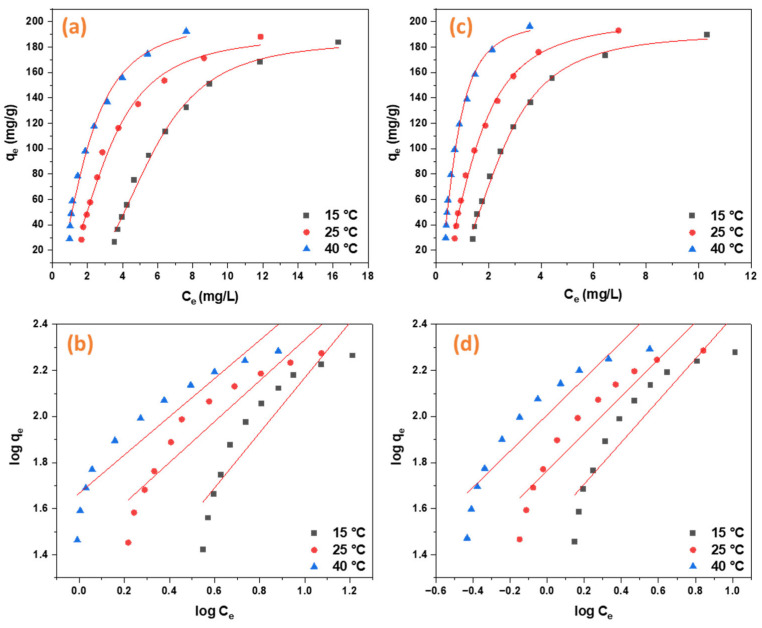
The Langmuir and Freundlich adsorption isotherms of CR and ORII. (**a**) CR Langmuir non-linear fit; (**b**) CR Langmuir linear fit; (**c**) ORII Freundlich non-linear fit; and (**d**) ORII Freundlich linear fit.

The adsorption data for both ORII and CR fit well with the Langmuir model, indicating that both were uniformly adsorbed onto the surface of the cellulose aerogel in a single molecular layer [54] (Figure 9). The adsorption of ORII and CR was observed to decrease with increasing initial dye concentrations. The Qmax for CR and ORII increases from 375.93 to 549.45 and from 129.87 to 171.23, respectively, as the temperature increases from 15 to 40 °C (Table 3). The significant interactions between the dye molecules and the available unoccupied active sites facilitated higher adsorption percentages at lower dye concentrations. The loading of the active sites and the repellent effect between the dye molecules and the overloaded active sites may explain why the removal percentage decreases with higher concentrations [3].

#### 2.2.4. Adsorption Thermodynamics

**Figure 10:**
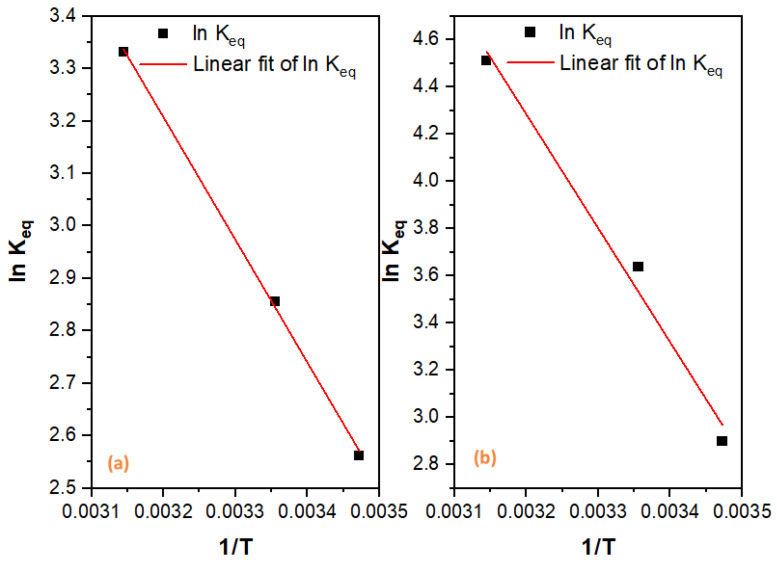
The linear diagram of the van’t Hoff equation to calculate the thermodynamic parameters (**a**) ORII and (**b**) CR.

The energetic and order driving forces behind adsorption behavior were investigated via thermodynamic studies at different temperatures (15, 25, and 40). The alterations in enthalpy (ΔH°), free energy (ΔG°), and entropy (ΔS°) were calculated and observed using the van’t Hoff equation (Table 4). An increased trend in the adsorption capacity of in situ UiO-66-NH_2_/TOCNF for both CR and ORII is noted with rising temperatures (Figure 10). The negative value of ΔG indicates that the adsorption reaction is spontaneous [55]. The absolute value of ΔG increases with increasing temperature, confirming that the driving force of the adsorption process is enhanced at higher temperatures, thereby facilitating adsorption. If the ΔS° value is positive, it indicates that the process is feasible since there is more variability in the adsorption reaction [56]. The adsorption process of CR and ORII using in situ UiO-66-NH_2_/TOCNF was endothermic, as shown by the positive ΔH° [57].

#### 2.2.5. Adsorption Mechanism

**Figure 11:**
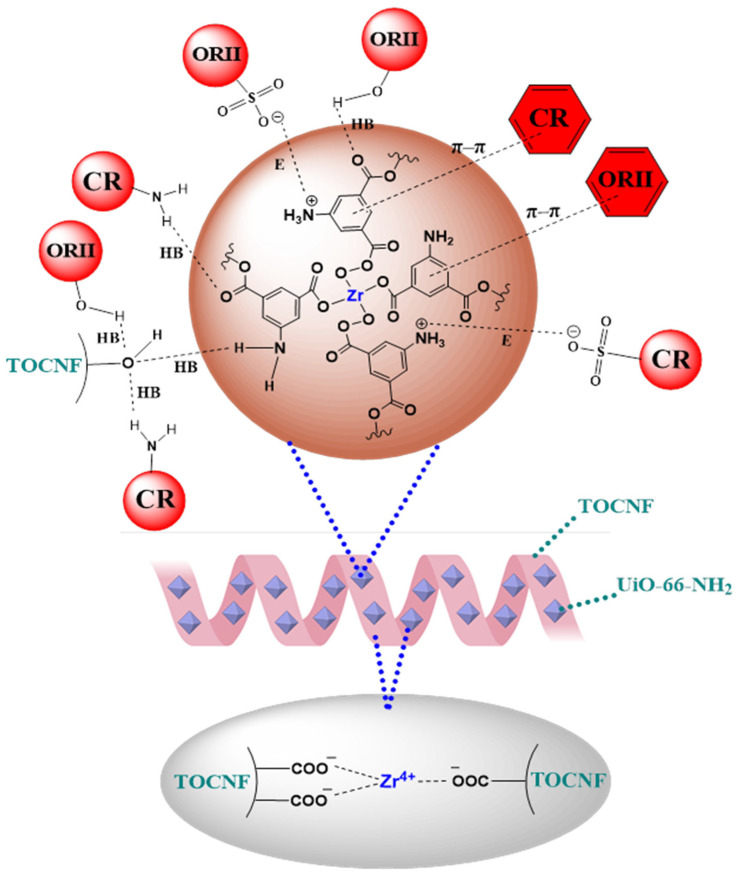
Schematic illustration of interactions between TOCNF and UiO-66-NH_2_ and adsorption interactions between dyes and UiO-66-NH_2_/TOCNF aerogel.

The presence of functional groups such as carboxyl and hydroxyl in TOCNF facilitates interactions with the amine groups and positively charged metal sites in UiO-66-NH_2_, resulting in strong MOF–fiber interactions and the formation of UiO-66-NH_2_/TOCNF composite structures. As shown in Figure 11, these interactions include hydrogen bonding between the hydroxyl and carboxyl groups in TOCNF and the primary amine and carboxyl groups in UiO-66-NH_2_, as well as coordination bonds between the carboxylate anions in TOCNF and the zirconium metal centers in UiO-66-NH_2_ [19]. The proposed adsorption mechanism of both dyes on the surface of the UiO-66-NH_2_/TOCNF aerogel is governed by three main interactions: (1) electrostatic attraction (E), where ammonium ions (–NH_3_⁺) on the adsorbent surface interact with the anionic sulfonic groups (–SO_3_^−^) of the dye molecules [58], along with the surface complexation between the Zr^4^⁺ nodes of UiO-66-NH_2_ and the –SO_3_^−^ functional groups of the dyes, leads to the formation of Zr–O–S bonds in both ORII and CR [59]; (2) hydrogen bonding, which occurs between the amine groups in CR, the hydroxyl groups of ORII and the hydroxyl, amine, and carboxyl groups present in UiO-66-NH_2_/TOCNF [60]; and (3) Van der Waals forces, including π–π stacking interactions between the aromatic rings of the dye molecules and the aromatic framework of the UiO-66-NH_2_/TOCNF aerogel [61].

#### 2.2.6. Regeneration Study

**Figure 12:**
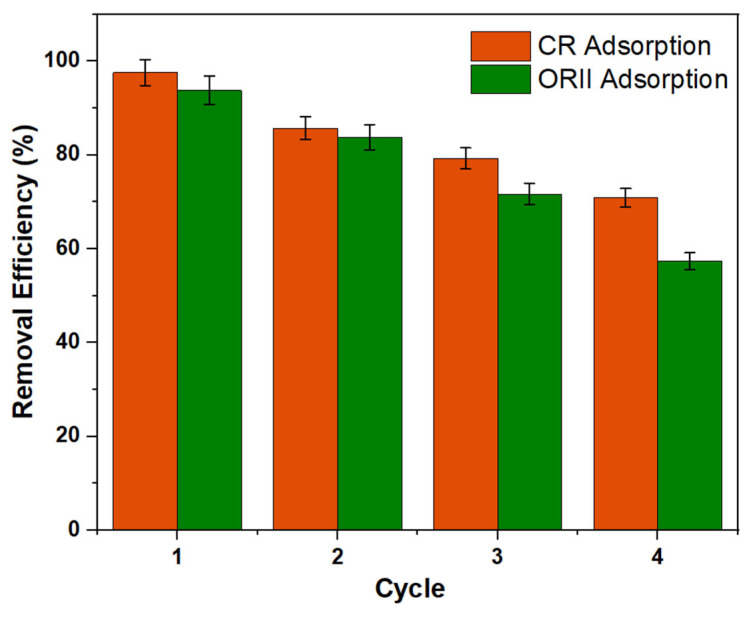
Regeneration of CR and ORII using in situ UiO-66-NH_2_/TOCNF.

The recycling and durability of adsorbents are crucial in real-world applications. Cellulose serves as a substrate for the growth of MOFs, enhancing their ability to absorb external stress effectively. The primary principle behind creating MOF and nanocellulose composites is to increase stability [18]. The SEM images revealed that even after CR and ORII have been adsorbed onto the surface of in situ UiO-66-NH_2_/TOCNF, the surface retains its initial form with minimal change, demonstrating excellent stability. We looked at the recyclability of the in situ adsorbent by comparing the total amount of dyes adsorbed after successive adsorption and extraction, via sodium hydroxide, cycles. The removal percentages of in situ UiO-66-NH_2_/TOCNF for CR and ORII remain at 70.84% and 57.33%, respectively, after four regeneration cycles, illustrating its effective recyclability (Figure 12). Overall, in situ UiO-66-NH_2_/TOCNF exhibited a comparatively higher maximum adsorption capacity than other adsorbents previously reported in the literature, as shown in Table 5. Although UiO-66-NH_2_, synthesized via the solvo-thermal method, resulted in comparatively higher maximum adsorption, it is a less environmentally compatible process and is prone to low robustness due to the lack of a matrix to support the particulate MOF.

## 3. Conclusions

In this study, we synthesized UiO-66-NH_2_, as well as stable and structured in situ UiO-66-NH_2_/TOCNF and ex situ UiO-66-NH_2_/TOCNF, using a TEMPO-oxidized cellulose nanofiber substrate for the removal of two azo dyes, CR and ORII. The in situ UiO-66-NH_2_/TOCNF exhibited the highest specific surface area and maximum adsorption capacity compared to the other two adsorbents, likely due to the uniform distribution of UiO-66-NH_2_ within the TOCNF matrix, which enhanced interface interactions, reduced the aggregation of MOFs, and provided a greater number of active sites for dye adsorption. The SEM imaging showed the growth of in situ UiO-66-NH_2_/TOCNF on the TEMPO-oxidized cellulose nanofiber substrate. Both CR and ORII demonstrated the highest adsorption capacities of 549.4 and 171.2 mg/g, respectively, at pH 2. The adsorption data fit a second-order and a Langmuir isotherm model, indicating that adsorption occurs via a chemisorption mechanism on a monolayer of the system.

## 4. Materials and Methods

### 4.1. Materials

Congo Red (CR, Sigma Aldrich, CAS-No. 573-58-0, empirical formula: C32H22N6Na2O6S2, MW: 696.66) was obtained from Sigma Aldrich (St. Louis, MO, USA). Orange II (ORII, Thermo Fisher Scientific, CAS 63396-5, empirical formula: C16H11N2NaO4S, MW: 350.324) was obtained from Thermo Fischer Scientific (Waltham, MA, USA). TEMPO-oxidized cellulose nanofiber suspension was obtained from the University of Maine. The TEMPO-oxidized cellulose nanofiber suspension was washed several times to remove the sodium salt. Ethanol > 99% pure, Zirconium (IV) chloride and 5-Aminoisophthalic acid (AIPA), and Epichlorohydrin (EPH), 99% were purchased from Thermo Fischer Scientific (Waltham, MA, USA). Deionized water of 17.8 megaohm was used for all experiments.

### 4.2. Preparation of UiO-66-NH_2_ and In Situ UiO-66-NH_2_/TOCNF

The UiO-66-NH_2_ was prepared by dissolving 1.17 g of ZrCl_4_ and 0.91 g of AIPA in 80 mL of ethanol. The mixture was transferred to an autoclave reactor and heated for 24 h at 120 °C. Afterward, the mixture was centrifuged, washed, and oven-dried for further use. The UiO-66-NH_2_/TOCNF composite aerogel was fabricated using in situ and ex situ techniques. For in situ UiO-66-NH_2_/TOCNF preparation, 1.17 g of ZrCl_4_ and 0.91 g of AIPA were dissolved in 140 mL of ethanol, followed by the addition of 30 mL of 3% TOCNF. The mixture was sonicated for 30 min, then transferred into an autoclave reactor and heated for 24 h at 120 °C. Subsequently, the mixture was centrifuged, washed, oven-dried, and freeze-dried for 3 days. In ex situ UiO-66-NH_2_/TOCNF preparations, 0.3 g of UiO-66-NH_2_ and 0.176 g of TOCNF aerogel were added to water until a good dispersion of the aerogel formed, followed by the addition of 7.8 mL of EPH. The mixture was then refluxed at 80 °C for 24 h. Finally, the mixture was centrifuged, washed, and freeze-dried for 3 days.

### 4.3. Aerogel Characterization

The physicochemical properties of in situ UiO-66-NH_2_/TOCNF, ex situ UiO-66-NH_2_/TOCNF, and UiO-66-NH_2_ were investigated through various means. The surface morphology was examined using FE-SEM (JEOL JSM-6500F, Tokyo, Japan) operating at 5 kV and equipped with energy dispersive X-ray (EDX) analysis. A thermal study (TGA-DTG) was conducted with the thermogravimetric analyzer (SDT Q600 series, TA instrument, New Castle, DE, USA). The surface area and pore volume of in situ UiO-66-NH_2_/TOCNF, ex situ UiO-66-NH_2_/TOCNF, and UiO-66-NH_2_ were measured using the Quantachrome Autosorb iQ gas sorption analyzer (Quantachrome ASIC05005, West Palm Beach, FL, USA). ATR-FTIR spectroscopy (Thermo Scientific Nicolet IS50 FTIR spectrometer, Ramsey, MN, USA) was performed at room temperature over the range of 400–4000 cm^−1^ to determine the functional groups in the different adsorbents.

### 4.4. Point of Zero Charge (PZC)

The pH drift technique was used to measure the PZC of in situ UiO-66-NH_2_/TOCNF. A series of 0.01 M NaCl solutions with varying pH values of 2, 4, 6, 10, and 12 was prepared using NaOH and HCl. N_2_ gas was utilized to eliminate dissolved CO_2_ from the NaCl solutions before adding in situ UiO-66-NH_2_/TOCNF. A 20 mg sample of in situ UiO-66-NH_2_/TOCNF was added to 20 mL of 0.01 M NaCl solution and placed on a stirrer for 24 h at 250 rpm. The solutions were filtered, and the final pH was measured after 24 h. The initial and final pH of the solutions were plotted in a graph to evaluate the PZC.

### 4.5. Adsorption Experiment

#### 4.5.1. pH Influence

The influence of pH on the adsorption capability of in situ UiO-66-NH_2_/TOCNF for removing CR and ORII (200 ppm) was examined by preparing a 10 mL solution at different pH ranges from 2 to 12. A 10 mg of in situ UiO-66-NH_2_/TOCNF was added to each vial and agitated for 75 min for CR and 2 h for ORII. After shaking, the samples were filtered using a 0.22 µm syringe filter.

#### 4.5.2. Adsorption Kinetics

The adsorption potential of all adsorbents (in situ UiO-66-NH_2_/TOCNF, ex situ UiO-66-NH_2_/TOCNF, and UiO-66-NH_2_) was initially studied using 10 mg of each adsorbent in 10 mL of CR and ORII (200 ppm) at pH 6 and 25 °C to optimize the adsorbent performance. The effect of different contact times was measured for the removal of CR and ORII using in situ UiO-66-NH_2_/TOCNF. In total, 10 mg of in situ UiO-66-NH_2_/TOCNF was added to 10 mL of CR and ORII solutions at concentrations of 100, 150, and 200 ppm. The experiments were conducted at pH 6, a temperature of 25 °C, and an agitation speed of 250 rpm, with contact times ranging from 1 to 300 min. After specific time intervals, the samples were removed from the shaker and filtered using a 0.22 µm syringe filter. The CR and ORII concentrations were measured before and after the adsorption experiment using a UV–Vis spectrophotometer (Azzota SM1800PC, Claymont, DE, USA) at wavelengths of 565 nm for CR and 365 nm for ORII. The removal efficiency and adsorption capacity of both dyes were calculated using the following formulas:

(1) $$\%\;\mathrm{removal}=\left(\frac{C_{0}-C_{e}}{C_{0}}\right)\times 100$$

(2) $$\mathrm{qt}=\frac{\left(C_{0}-C_{t}\right)V}{m}$$

The initial concentration is C_0_ (mg/L), the dye concentration at time t is represented by Ct (mg/L), V is the volume (L), and the adsorbent mass is expressed by m (g).

#### 4.5.3. Adsorption Isotherms

The adsorption capacity of in situ UiO-66-NH_2_/TOCNF for removing CR and ORII at different concentrations and varying temperatures was studied. In total, 10 mg of in situ UiO-66-NH_2_/TOCNF was added to 10 mL of CR and ORII solutions with concentrations ranging from 20 to 200 ppm and a pH of 6. The samples were placed on a shaker at three different intervals, with temperatures of 15, 25, and 40 °C. At the end, the samples were filtered using a 0.22 µm syringe filter, and the remaining concentrations of both dyes were analyzed using UV/Vis spectrophotometry. The final values were evaluated by fitting the data into Langmuir and Freundlich isotherms. The equations for both adsorption isotherms and adsorption kinetics are presented in Table 6.

$C_{e}$ (mg/L) is the dye concentration at equilibrium, $q_{e}$ (mg/g) is the equilibrium adsorption capacity, $k_{1}$ (min^−1^) is the Langmuir adsorption constant related to adsorption energy, and $k_{2}$ (g/(mg min)) is the rate constant of pseudo-second order adsorption, $q_{max}$ (mg/g) is the maximum adsorption capacity, $q_{t}$ (mg/g) is the adsorption capacity at time t, k_1_ (min^−1^) is the rate constant of pseudo-first order adsorption, and $k_{f}$ and n are the Freundlich adsorption constants signifying the capacity and intensity of the adsorption, respectively.

#### 4.5.4. Adsorption Thermodynamics

The influence of temperature on the internal energy of the in situ UiO-66-NH_2_/TOCNF was studied. A 10 mg sample of in situ UiO-66-NH_2_/TOCNF was added to 10 mL of CR and ORII (200 ppm) solutions at pH 6 and placed on a shaker at three different temperatures (15, 25, and 40 °C). The concentration of dye filtrate was measured using UV/Vis spectrophotometry. The obtained values were analyzed using van’t Hoff equations to determine the changes in adsorption-related thermodynamic parameters.

(3) $$\Delta G=-{\mathrm{RTlnK}}_{\mathrm{eq}}$$

(4) $${\mathrm{lnK}}_{\mathrm{eq}}=\frac{\Delta S}{R}-\frac{\Delta H}{\mathrm{RT}}$$

(5) $$K_{\mathrm{eq}}=\frac{q_{e}}{\begin{array}{c} C_{e} \end{array}}$$

In these equations, R is the gas constant (8.314 J K^−1^), ΔH is the change in enthalpy (KJ·mol^−1^), ΔG is the standard free energy change (kJ·mol^−1^), ΔS is the change in entropy (J·mol^−1^ K^−1^), and K_eq_ is the equilibrium constant, heat capacity, and distribution coefficient, respectively.

### 4.6. Reusability Study

The recyclability of in situ UiO-66-NH_2_/TOCNF for the adsorption of CR and ORII was studied over four cycles. A total of 10 mg of in situ UiO-66-NH_2_/TOCNF was added to 10 mL of 200 ppm CR and ORII solutions at pH 6 and a temperature of 25 °C, then placed on a shaker for 75 min and 2 h, respectively. After each adsorption cycle, the used in situ UiO-66-NH_2_/TOCNF was immersed in 10 mL of 0.1 M NaOH solution and agitated for 4 h at 250 rpm. After filtering, the adsorbent was washed 2 to 3 times before the next cycle to neutralize it and was then dried.

## Figures and Tables

**Table 1:** BET surface area of UiO-66-NH_2_, in situ UiO-66-NH_2_/TOCNF, and ex situ UiO-66-NH_2_/TOCNF.

Materials	BET (m^2^/g)	Pore Volume cc/g
UiO-66-NH_2_	60.42	0.23
in situ UiO-66-NH_2_/TOCNF	145.67	0.39
ex situ UiO-66-NH_2_/TOCNF	26.70	0.15

**Table 2:** Adsorption kinetic parameters for CR and ORII at an initial concentration of 100, 150, and 200 mg/L, a dosage of 10 mg/L, and a temperature of 25 °C.

Models		CR			ORII		
	C_o_ (mg/L)	100	150	200	100	150	200
Pseudo−first order	K_1_ (1/min)	0.04708	0.03646	0.03777	0.03771	0.03364	0.03849
	q_e_ (mg/g)	72.67083	122.5097	176.7524	78.22427	113.0274	147.831
	R^2^	0.96442	0.98535	0.96711	0.9884	0.98676	0.99391
Pseudo−second order	K_2_ (g/mg/min)	0.001701	0.000493	0.000374	0.000592	0.000251	0.000303
	q_e_ (mg/g)	101.626	156.9859	208.7683	100.5025	160.5136	204.0816
	R^2^	0.99954	0.99816	0.99788	0.9976	0.99453	0.99804

**Table 3:** Langmuir and Freundlich isotherm model values for CR and ORII at an initial concentration of 20 to 200 (mg/L) and three different temperatures (15, 25, and 40 °C).

Models		CR			ORII		
	Temp (°C)	15	25	40	15	25	40
Langmuir	K_1_ (L/mg)	0.384949	0.186695	0.06587	1.915423	0.115305	0.761408
	q_max_ (mg/g)	375.9398	383.1418	549.4505	129.8701	148.8095	171.2329
	R^2^	0.99345	0.99554	0.99604	0.99976	0.98957	0.99894
Freundlich	K_F_ ([mg g^−1^(Lmg^−1^)1/n])	101.763	58.13933	33.55135	84.30629	26.87571	74.98424
	n	1.269728	1.237517	1.103107	12.51251	3.246964	6.436663
	R^2^	0.85886	0.87461	0.83526	0.90941	0.99024	0.94508

**Table 4:** The distribution and thermodynamic parameters calculated for the adsorption of CR and ORII at different temperatures.

Adsorbate	T (°C)	Q_e_ (mg/g)	$K_{d}=\frac{q_{e}}{C_{e}}$	$lnK_{d}$	ΔG° (KJ·mol^−1^)	ΔH° (KJ·mol^−1^)	ΔS° (J·mol^−1^·K^−1^)
CR	15	189.57	18.17546	2.90007	−6.94403	40.15255	164.0981
	25	194.87	37.98635	3.63722	−9.01149		
	40	197.83	91.1659	4.51268	−11.9309		
ORII	15	185.68	12.96648	2.56236	−6.13541	19.4291	88.83509
	30	189.13	17.39926	2.85642	−7.07701		
	40	193.1	27.98551	3.33168	−8.80849		

**Table 5:** Comparison of the in situ UiO-66-NH_2_/TOCNF adsorption capacities for CR and ORII with other adsorbents reported in the literature.

Adsorbent Material	Dye Type	Adsorption Capacity (mg/g)	References
Orange II			
UiO-66-NH_2,_ solvothermal method	ORII	229.8	[44]
CSSA Hydrogel	ORII	6.84	[62]
Apricot shell activated carbon	ORII	13.98	[63]
Canola stalks	ORII	25.6	[64]
Zn_2_Al-layered double hydroxide	ORII	42.5	[65]
cetyltrimethylammonium bromide (CTAB)	ORII	110.05	[66]
ZnO-modified g-C_3_N_4_ composite	ORII	13.441	[67]
In situ UiO-66-NH_2_/TOCNF	ORII	171.2	This study
Congo Red			
UiO-66-NH_2_@HTC	CR	277.77	[23]
ZnCuCr-Based MOF	CR	325	[68]
[Ni_2_F_2_(4,4′-bipy)_2_(H_2_O)_2_](VO_3_)_2_.8H_2_O	CR	242.1	[69]
UiO-66-NH_2_]	CR	16.50	[20]
Iron oxide/carbon composite	CR	40.44	[70]
Polycationic Fe/Al Di-metal nanostructured composite (PDFe/Al)	CR	411	[71]
CS/PEG/ZnO Composite Hydrogel	CR	212.76	[72]
Nylon fiber waste	CR	188	[73]
Activated biochar	CR	114.8	[74]
Fly-Ash@Fe_3_O₄	CR	154	[75]
In situ UiO-66-NH_2_/TOCNF	CR	549.4	This study

**Table 6:** Adsorption and kinetics study models.

Model	Equation
Langmuir linear	$\frac{C_{e}}{q_{e}}=\frac{1}{K_{l}{\;q}_{\max}}+\frac{C_{e}}{q_{\max}}$
Langmuir non-linear	$q_{e}=\frac{q_{m}K_{l}C_{e}}{1+K_{l}C_{e}}$
Freundlich linear	$\ln\left(q_{e}\right)=\left[\ln\left(K_{f}\right)+\left(\frac{1}{n}\right)\ln\left(C_{e}\right)\right]$
Freundlich non-linear	$q_{e}=k_{f}{C_{e}}^{1/n}$
Pseudo-first order non-linear	$q_{t}=q_{e}\left(1-e^{-k_{1}t}\right)$
Pseudo-first order linear	$\log\left(q_{e}-q_{t}\right)=\left[\log\left(q_{e}\right)-\left(\frac{k_{1}}{2.303}\right)\times t\right]$
Pseudo-second order non-linear	$q_{t}=\frac{k_{2}{{\mathrm{tq}}_{e}}^{2}}{1+k_{2}{\mathrm{tq}}_{e}}$
Pseudo-second order linear	$\frac{t}{q_{t}}=\frac{1}{k_{2}{\left(q_{e}\right)}^{2}}+\frac{t}{q_{e}}$

## Data Availability

Data are contained within the article.
